# Four new species of Sapindaceae from the Guianas

**DOI:** 10.3897/phytokeys.7.1956

**Published:** 2011-11-29

**Authors:** Pedro Acevedo-Rodríguez

**Affiliations:** 1 Department of Botany, MRC-166 Smithsonian Institution, P.O. Box 37012, Washington D.C. 20013-7012, USA

**Keywords:** Guyana, French Guiana, *Matayba ayangannensis*, *Paullinia degranvillei*, *Paullinia oldemanii*, *Paullinia prevostiana*, Sapindaceae, South America

## Abstract

Four new species of Sapindaceae from the Guianas, South America, are described, illustrated and contrasted with their putative, relatives: *Matayba ayangannensis* Acev.-Rodr. a small shrub from Mt. Ayanganna, Guyana; and *Paullinia degranvillei* Acev.-Rodr., *Paullinia oldemanii* Acev.-Rodr., and *Paullinia prevostiana* Acev.-Rodr., three species of lianas from French Guiana.

## Introduction

While working on a treatment-sec of Sapindaceae for the Flora of the Guianas (Acevedo-Rodríguez in prep.) four new species were discovered, one in *Matayba*, and three in *Paullinia*. In the Neotropics, Sapindaceae is a ubiquitous plant family with 37 genera and about 800 species. It is characterized by a woody habit (either trees, shrubs or lianas), compound alternate leaves, and flowers with petals usually bearing petaloid appendages on the adaxial surface. Fruit type and morphology are rather variable and often the basis for generic delimitation. *Matayba* is a Neotropical genus with about 50 species of trees or shrubs, 11 of which are found in the Guianas. *Paullinia*, also restricted to the Neotropics (except for *Paullinia pinnata*, which is found also in Africa and Madagascar), has about 200 species of lianas or climbing shrubs. *Paullinia* is the largest genus of Sapindaceae in the Guianas, with 39 species occurring there.

## Taxonomic treatment

### Matayba ayangannensis

Acev.-Rodr., sp. nov.

urn:lsid:ipni.org:names:77115892-1

http://species-id.net/wiki/Matayba_ayangannensis

Fig. 1A–I 

#### Latin

Frutex 2.5 metralis; foliis 4–6 foliolatis; foliolis alternis vel sub oppositus, discolores, ellipticus, apice rotundatis retusisque; calyce tomentelli, petalis obovatis.

#### Type.

Guyana. Cuyuni-Mazaruni. Mt. Ayanganna, S.S. Tillet, C.L. Tillet, & R. Boyan 45080 (holotype: NY!; isotypes: MO!, US!).

#### Description.

Shrub to 2.5 m tall. Stems glabrous, striate. Leaves paripinnate; petiole plus rachis 2–6.5 cm long, slightly flattened adaxially, striate, puberulent; petiolules ca. 5 mm long, pulvinate at base; leaflets (4) 6, 2.5–4.5 × 1.2–2.3 cm, opposite or sub-opposite, obovate, oblanceolate or nearly elliptic, rigidly coriaceous, brittle, discolorous (abaxial surface drying brownish), adaxially glabrous, abaxially puberulent, especially along midvein, the base obtuse, symmetrical, the apex emarginate or less often rounded, the margins entire, slightly revolute; abaxially the midvein prominent, secondary and tertiary veins inconspicuous, reticulate. Thyrses 8–18 cm long, axillary, on distal portion of branches, paniculate, with ferruginous-pubescent and slightly angled axes. Flowers in simple dichasia; pedicels ca. 2 mm long, pubescent. Calyx brownish yellow, ferruginous-pubescent, ca. 1 mm long, the lobes 0.5–0.7 mm long, ovate; petals ca. 2 mm long, yellowish white, obovate, emarginate at apex, lanose mostly along margins; appendages 2, ca. 1 mm long, sericeous-tomentose, supra basal; disc glabrous, pulvinate; stamens 2–2.5 mm long, the filaments lanose on lower half; ovary tomentulose, the style subcapitate. Capsules not known.

#### Distribution and ecology.

Known only from the type collection in Guyana, in low forest.

#### Discussion.

*Matayba ayangannensis* looks vegetatively similar to *Matayba yutajensis* Steyerm., as both species are shrubs and have leaflets with retuse apex and reticulate venation; however,  *Matayba ayangannensis*differs from the latter by its leaves with (4) 6, discolorous, brittle leaflets that are 2.5–4.5 cm long (vs. 2(4), concolorous, coriaceous, 10–18 cm long), and by its ferruginous-pubescent calyx, ca. 1 mm long (vs. glabrous or sparsely strigose at base and ca. 3.5 mm long).

#### Etymology.

The epithet refers to Mt. Ayanganna where the type collection was made.

**Figure 1.**
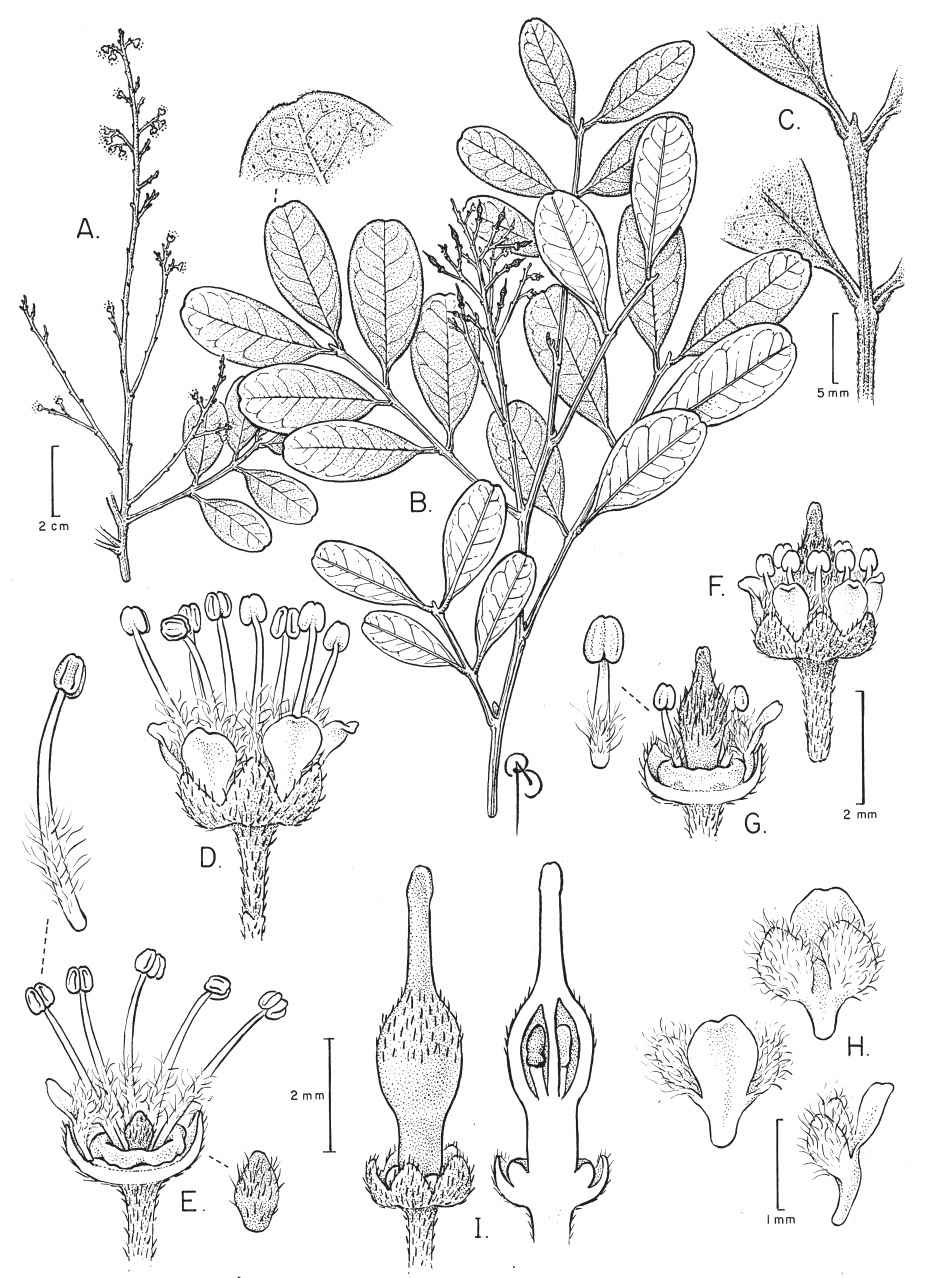
*Matayba ayangannensis* **A** Branch with staminate flowers **B** Branch with pistillate flowers **C** Distal portion of leaf rachis showing distal process and bases of 4 leaflets **D** Staminate flower **E** Staminate flower with part of perianth removed to show nectary disc, stamens and pistillode; detail of pistillode **F** Pistillate flower **G** Pistillate flower with part of perianth removed to show nectary disc and gynoecium; detail of staminode **H** Abaxial, adaxial and lateral views of petal with appendages **I** Pistillate flower with partly developed gynoecium, l. s. of same. All from Tillet 45080 (NY).

### Paullinia degranvillei

Acev.-Rodr., sp. nov.

urn:lsid:ipni.org:names:77115893-1

http://species-id.net/wiki/Paullinia_degranvillei

Fig. 2 

#### Latin

A Paullinia obovata (Ruiz & Pav.) Pers. folioliis nervo reticulatis e fructo pyriformes diversa.

#### Type.

French Guiana. Crique Caiman, rive gauche de l’ Oyapock à environ 70 km SW St. Georges, de Granville T-1065 (holotype: CAY!; isotypes: CAY!, P, US!).

#### Description.

Liana to 30 m long. Stems terete, lenticellate, ferruginous, minutely sericeous-pubescent; cross section of stem with a single vascular cylinder. Stipules ca. 0.5 mm long, triangular, early deciduous. Leaves pinnately 5-foliolate; petioles (1) 7–9 cm long; unwinged, adaxially furrowed, sericeous-pubescent to glabrous; rachis 2–8 cm long, narrowly winged or margined, sericeous-pubescent to glabrous; petiolules of distal and lateral leaflets 2–2.5 cm long, glabrous; leaflets (3.5) 12–22 × (2.7) 5.5–8 cm, elliptic, coriaceous, glabrous, the base acute or obtuse on lateral and basal leaflets, attenuate on distal leaflet, the apex obtusely acuminate to caudate, glandular at the very tip, the margins crenate or remotely glandular-dentate, revolute; venation prominent abaxially; tertiary venation reticulate. Thyrses 3–23 cm long, axillary, solitary, racemiform, with sericeous-pubescent axes; cincinni sessile, few-flowered. Flowers unknown (remnant sepals sericeous). Capsule 4–5 cm long, woody, pyriform, unwinged, reddish, long-stipitate (5–8 mm long), glabrous, pericarp ca. 4 mm thick, endocarp sparsely pubescent or glabrous. Seed unknown.

#### Distribution and ecology.

Known only from French Guiana, from submontane forest.

#### Additional specimen examined.

French Guiana**.** Monts Atachi Bacca, 780 m elev., de Granville et al. 10833 (B, CAY, US).

#### Discussion.

*Paullinia degranvillei* seems to be closely related to *Paullinia obovata* (Ruiz & Pav.) Pers. as they both have large, unwinged capsules with a thick mesocarp. *Paullinia degranvillei* differs from *Paullinia obovata* by it leaflets with tertiary reticulate venation (vs. clathrate) and by its pyriform fruits (vs. obovoid).

#### Etymology.

The specific epithet honors Dr. Jean-Jacques de Granville, who collected the type of this new species.

**Figure 2.**
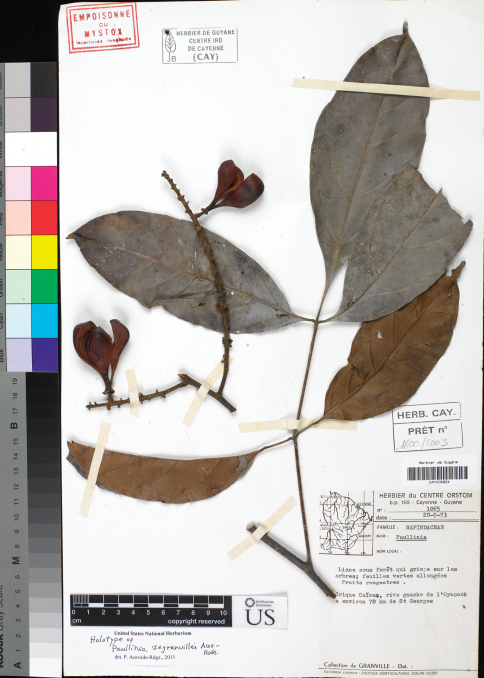
*Paullinia degranvillei*. Photo of the holotype.

### Paullinia oldemanii

Acev.-Rodr., sp. nov.

urn:lsid:ipni.org:names:77115894-1

http://species-id.net/wiki/Paullinia_oldemanii

Fig. 3A–J 

#### Latin

*A Paullinia venosa* Radlk. *foliis menores, foliis rachides alatis et foliolis distalis obovatis diversa.*

#### Type.

French Guiana. Fleuve Oyapock, 300 m après le camp saut Moutouci, Oldeman B-3223 (holotype: US; isotype: CAY).

#### Description.

Liana to 10 m long. Stems terete, minutely sericeous-pubescent, lenticellate; cross section with a single vascular cylinder. Stipules ca. 1mm long, deltate, early deciduous. Leaves 5-pinnate foliolate; petioles 1–5 cm long, unwinged, glabrous; rachis 1–1.8 cm long, narrowly winged or margined, glabrous; leaflets 6–9.5 × 2–3.5 cm, chartaceous, elliptic, oblong, or rarely lanceolate, glabrous except for the minute hairy domatia in the secondary vein axils, distal leaflet obovate, the base decurrent on distal leaflet, obtuse on laterals, and obtuse to truncate on proximal ones, the apex obtusely acuminate to nearly caudate, margins sinuate, the lateral and proximal leaflets with a glandular tooth on both margins near the base; tertiary venation finely reticulate. Thyrses 7–13 cm long, axillary, racemiform, solitary, the axes ferruginous-tomentulose; cincinni sessile, few-flowered; bracts and bracteoles minute, deltate. Pedicels 1.5–2 mm long, articulate near the middle, ferruginous-tomentulose. Calyx sparsely appressed-pubescent, sepals 5, concave, with ciliate-glandular margins, the outer ones ca. 1.5 mm long, ovate to rounded, the inner ones 3–4 mm long; petals ca. 4 mm long, oblong, rounded at apex, shortly clawed at base, adaxially papillate; appendages as long as the petals, crest of posterior appendages with 2 corniform projections at apex, lateral ones with 1 corniform projection, these ca. 1.7 mm long; nectary with 2 deltate, pubescent lobes ca. 1 mm tall; filaments 2–4 mm long, of unequal lengths, pubescent on lower half; anthers ca. 1 mm long, ellipsoid; gynoecium ferruginous-tomentulose, clavate, with three stigmatic branches. Capsule (immature) unwinged, long-stipitate, smooth, nearly globose.

#### Distribution and ecology.

Known from French Guiana and Brazil (Amazonas), in riverside and moist forests.

#### Additional specimens examined.

French Guiana**.** Oyapok River, Oldeman B-3418 (MO), Saut Kouamantapéré, Oldeman T-662 (CAY); Saut Tainoua, riverside forest, de Granville 387 (CAY, MO, US). Brazil. Amazonas; ca. 4 kn NW of Balbina dam, Thomas et al. 5354 (NY).

#### Discussion.

*Paullinia oldemanii* seems to be closely related to *Paullinia venosa* Radlk. as they share similar vegetative and fruit morphologies. *Paullinia oldemanii* however differs from *Paullinia venosa* by its abaxially puberulent leaflets 1.7–7 cm long (vs. glabrous and 3.5–17 cm long), distal leaflets obovate (vs. oblong or lanceolate), leaf rachis narrowly winged or margined (vs. terete, unwinged) and its calyx of 5 sepals (vs. 4 sepals).

#### Etymology.

The specific epithet honors Professor Dr. R.A. Oldeman, collector of the type specimen.

**Figure 3.**
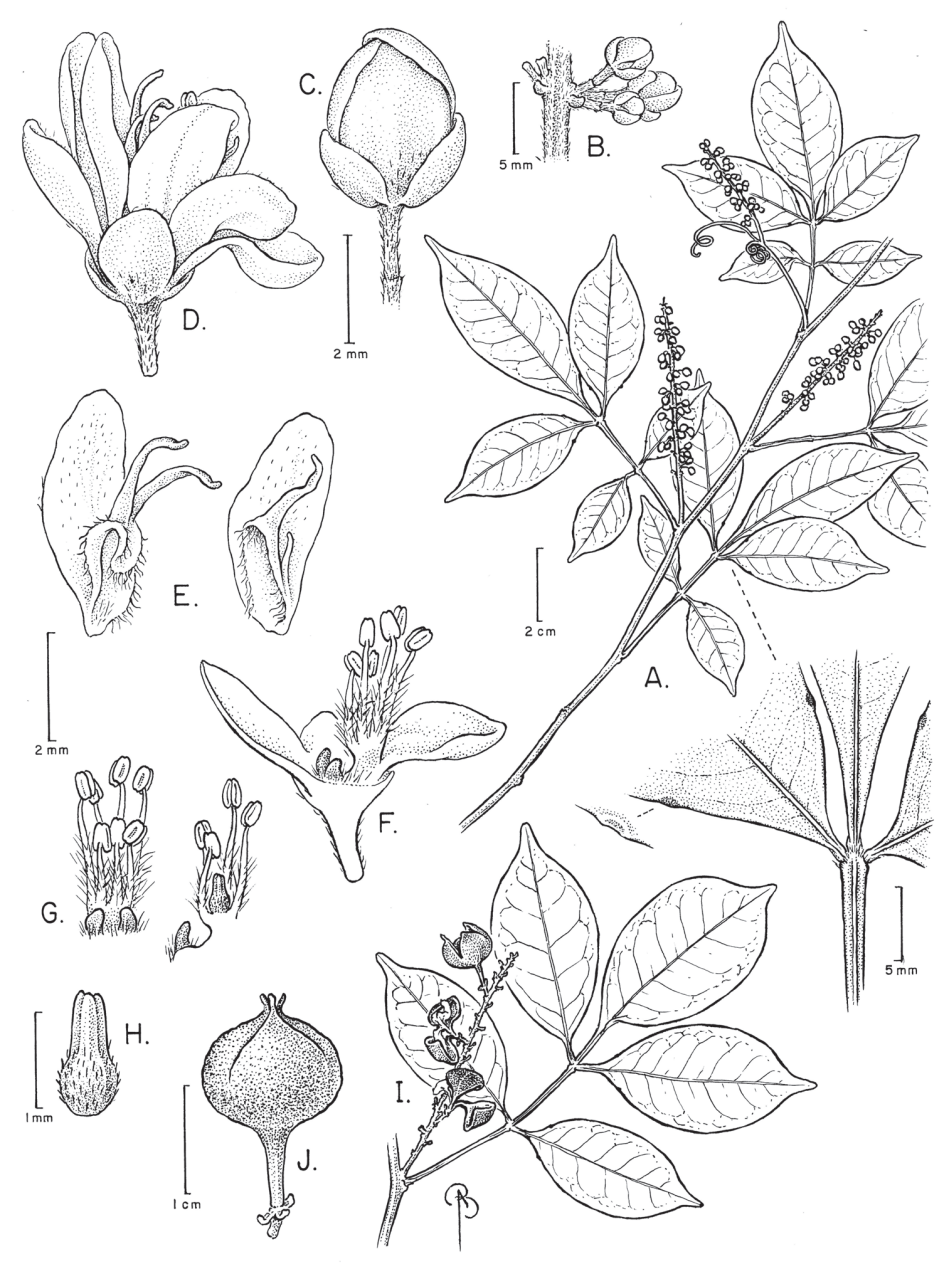
*Paullinia oldemanii* **A** Flowering branch, with detail of distal portion of leaf, showing glandular teeth near the base of lateral leaflets **B** Cincinnus **C** Flower bud **D** Staminate flower, lateral view **E** Posterior and lateral petals with adnate appendages **F** Staminate flower with part of perianth removed, showing nectary lobes and stamens **G** Stamens and nectary lobes, anterior and lateral views; H Pistillode **I** Fruiting branch **J** Fruit. **A-H** from Oldeman B-3223 (US),; **I-J** from Thomas et al. 5354 (NY).

### Paullinia prevostiana

Acev.-Rodr., sp. nov.

urn:lsid:ipni.org:names:77115895-1

http://species-id.net/wiki/Paullinia_prevostiana

Fig. 4A–I 

#### Latin

A paullinia tenuifolia Standl. foliis magni, hispidulosi; rachides tereti, hispidulosi; sepaliis exteriores minoriis diversa.

#### Type.

French Guiana. Piste de St. Elie, primary forest, 5 17N; 53 3W, 6 Nov 1999, Prévost 3741 (holotype: US!; isotype: CAY!).

#### Description.

Slender, woody vine 15–30 m long. Stems terete, striate, hispidulous, becoming glabrous, cylindrical, and lenticellate with age, not producing milky sap; cross section of stem with a single vascular cylinder. Stipules 5–12 mm long, subulate or linear, hispidulous. Leaves pinnately 5-foliolate; petiole and rachis unwinged, hispid; petioles 3.5–10 cm long; rachis 3–6 cm long; leaflets 5–18 × 3.5–9 cm, ovate, elliptic, oblong-elliptic, or obovate, chartaceous, upper surface glabrous except for the pubescent to tomentose mid and secondary veins, lower surface sparsely pilose, especially on veins, the base obtuse to rounded on lateral leaflets, cuneate or acuminate on distal leaflets, the apex acuminate to long acuminate, the margins remotely dentate with glandular teeth, ciliate; tertiary venation clathrate. Thyrses ca. 10 cm long, without tendrils, cauliflorous, fasciculate, with hispidulous axes; cincinni sessile, opposite, 4- to 5-flowered. Pedicels 4.5–6 mm long, articulate near the base. Calyx light green, appressed-pubescent, sepals 5, concave, rounded at apex, the outer sepals 1.5–2 mm long, ovate, the inner sepals 3–3.5 mm long; petals ca. 5 mm long, white, oblong, slightly asymmetrical, shortly clawed at base, rounded at apex, papillate on both surfaces; appendages ca. 4 mm long, crest of posterior appendages with 2 corniform, fleshy projections at apex, the ligule sub-lanose; disc 4-lobed, glabrous, the posterior lobes ovate, dorsally flattened, the lateral lobes similar, but smaller than the posterior ones; torus hispidulous; filaments 4–7 mm long, of unequal lengths, flattened, densely lanose; anthers ca. 0.7 mm long. Capsules ca. 15 mm long, fusiform, sometimes keeled or narrowly winged along dorsal side of pericarp, ferruginous, short-pilose, stipitate at base; endocarp wooly-pubescent. Seed obovoid, dark brown, glabrous, with a sarcotesta on lower ⅓, the sarcotesta dorsally notched.

#### Distribution and ecology.

Known only from Guyana and French Guiana, in dense, humid forests, to be expected in Suriname.

#### Additional specimens studied.

Guyana: Bartica. Linder 152 (NY). French Guiana: Camopi River, summit of Alikéné Mt., Oldeman & Sastre 301 (MO, US-2). Crique Grégoire, Deward 129 (CAY), savanna, Girard 189 (CAY). Noragues Field Station, Petit Plateau, ca. 200 m elev., Mori et al. 25555 (CAY, NY, US), forest at base of inselberg, Larpin 1035 (CAY-2, US). Région de Cayenne. south of Mt des Chevaux, Cremers 5310 (CAY).

#### Discussion.

*Paullinia prevostiana* seems to be closely related to *Paullinia tenuifolia* Standl. as they share the following characters: Leaves pinnately 5-foliolate, terete stems with a single vascular cylinder; and cauliflorous inflorescence. *Paullinia prevostiana* differs however by its hispid leaves (vs. puberulent or glabrous), leaflets 5–18 cm long (vs. 3.5–11 cm) and its outer sepals much shorter than the inner ones (vs. nearly as long as the inner ones).

#### Etymology.

The specific epithet honors Dr. Marie Françoise Prévost, plant ecologist at IRD who has collected numerous plant specimens from French Guiana, including the type of this species.

**Figure 4.**
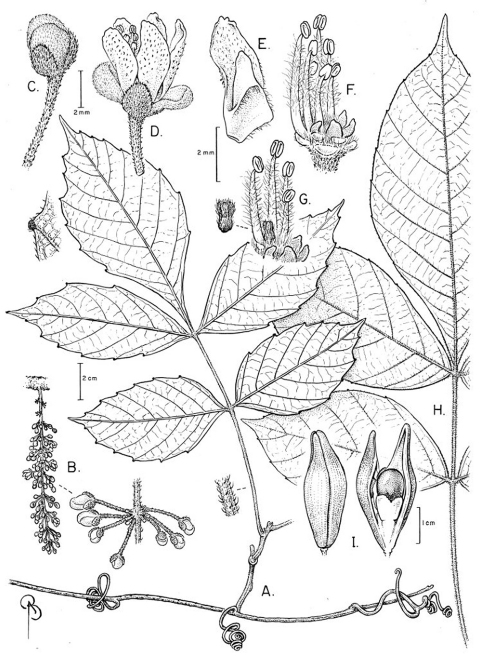
*Paullinia prevostiana* **A** Branch with leaf and tendrils, detail of marginal tooth, and detail of petiole pubescence **B** Inflorescence and detail of cincinnus **C** Flower bud **D** Staminate flower, lateral view **E** Lateral petal with appendage **F** Staminate flower with perianth removed to show nectary glands and stamens **G** Staminate flower with perianth and few stamens removed to show nectary glands and pistillode, detail of pistillode **H** Larger leaf **I** Capsule, un-dehisced and dehisced showing seed, basal sarcotesta, and funiculus. **A-G** from Prevost 3741 (US); **H-I** from Mori et al. 25555 (NY).

## Supplementary Material

XML Treatment for Matayba ayangannensis

XML Treatment for Paullinia degranvillei

XML Treatment for Paullinia oldemanii

XML Treatment for Paullinia prevostiana
